# Pyroptosis-Mediated Molecular Subtypes and Tumor Microenvironment Infiltration Characterization in Colon Cancer

**DOI:** 10.3389/fcell.2021.766503

**Published:** 2021-11-10

**Authors:** Jiawei Rao, Wen Li, Chuangqi Chen

**Affiliations:** ^1^Department of Colorectal Surgery, Gastrointestinal Surgery Center, The First Affiliated Hospital of Sun Yat-sen University, Guangzhou, China; ^2^Surgical Laboratory, The First Affiliated Hospital of Sun Yat-sen University, Guangzhou, China

**Keywords:** pyroptosis, colon cancer, tumor microenvironment, prognosis, therapy

## Abstract

The role of pyroptosis, which is also a kind of cell-intrinsic death mechanism, in tumorigenesis and cancer progression has been revolutionized. However, the expression of pyroptosis-related genes (PYGs) in colon cancer (CC) and their prognostic value remain unclear. In this study, we comprehensively identified two PYG-mediated molecular subtypes with a distinct tumor microenvironment (TME) in 1,415 CC samples, which were based on 10 PYGs. The six-gene signature (pyroptosis score, PY-score) was constructed to quantify the molecular patterns of individual tumors using a least absolute shrinkage and selection operator (LASSO)–Cox regression model through the differentially expressed genes between the two molecular subtypes. Significant infiltration of activated immune cells (such as M1 macrophages and cytotoxic T cells) was observed in the low PY-score group, while naive and suppressive immune cells (such as naive CD8^+^ T cells and M2 macrophages) dominated in the high PY-score group. CC patients in the low PY-score group showed not only significant survival advantage but also sensitivity to immune checkpoint inhibitor treatment, anti-epidermal growth factor receptor (EGFR) therapy, and chemotherapy. Overall, this work revealed that the PYGs played a vital role in the formation of heterogeneity in the TME. The analysis of the PYG-mediated molecular patterns helps in understanding the characterization of TME infiltration and provides insights into more effective therapeutic strategies.

## Introduction

As the third most common cancer worldwide, colon cancer (CC) deprived nearly 576,000 lives in 2020, according to related statistics (Global Cancer Observatory, 2021). Although remarkable progression has been made in the treatment of CC patients, the prognosis of patients with advanced stage remains unpromising. At present, the tumor node metastasis (TNM) staging system and other clinicopathological features are commonly used in prognostic stratification and the formulation of treatment strategies (Hu et al., 2011). However, some patients with the same TNM stages were reported to have distinct survival outcomes and variable responses to treatment (Nagtegaal et al., 2011; Zhai et al., 2017), which indicated the vital role of molecular and genetic alterations in cancer progression (Sirinukunwattana et al., 2021). Thus, an in-depth understanding of the molecular subtypes of CC patients may provide further prognosis prediction and, thus, effective treatment methods.

It is recognized that comprehensive regulation of cell-intrinsic death mechanisms, such as apoptosis and pyroptosis, takes part in the growth, invasion, and metastasis of cancer cells (Galluzzi et al., 2018). Compared with the widely known apoptosis, the role of pyroptosis in cancer occurrence and progression has only been revolutionized in the past few years. It was reported that the decreased expression of a pyroptosis-related gene (PYG), *GSDMD*, significantly induced the proliferation of gastric cancer cells *in vivo* and *in vitro* (Wang et al., 2018). Wang et al. (2019) also showed that metformin could promote the *GSDMD*-mediated pyroptosis in esophageal squamous cell carcinoma, which indicated the treatment potential of metformin. *GSDME*, which is an executor of cell pyroptosis, was also found to serve as a tumor suppressor and be silenced in several cancers (Wang et al., 2017). A recent research pointed out that combining decitabine with chemotherapy could trigger the pyroptosis of malignant colon cells and strengthen the immunological effects (Fan et al., 2019), which further confirmed the potential role of PYGs in prognostic stratification and the treatment targets of CC.

In this study, we developed two distinct molecular subtypes based on PYGs and analyzed the correlation between the subtypes and the components of their tumor microenvironment (TME) through the integration of the genomic data from 1,415 samples in the Gene Expression Omnibus (GEO) and The Cancer Genome Atlas (TCGA) datasets. For precise prognostic prediction of individual CC patients, we calculated the pyroptosis score (PY-score), which also helped predict the responses to immune checkpoint inhibitor (ICI) treatment, anti-epidermal growth factor receptor (EGFR) treatment, and chemotherapy. These findings suggested that PYGs could provide prognostic stratification and guide the treatment decisions for CC patients.

## Materials and Methods

### Source and Preprocessing of Colon Cancer Data

**FIGURE 1:**
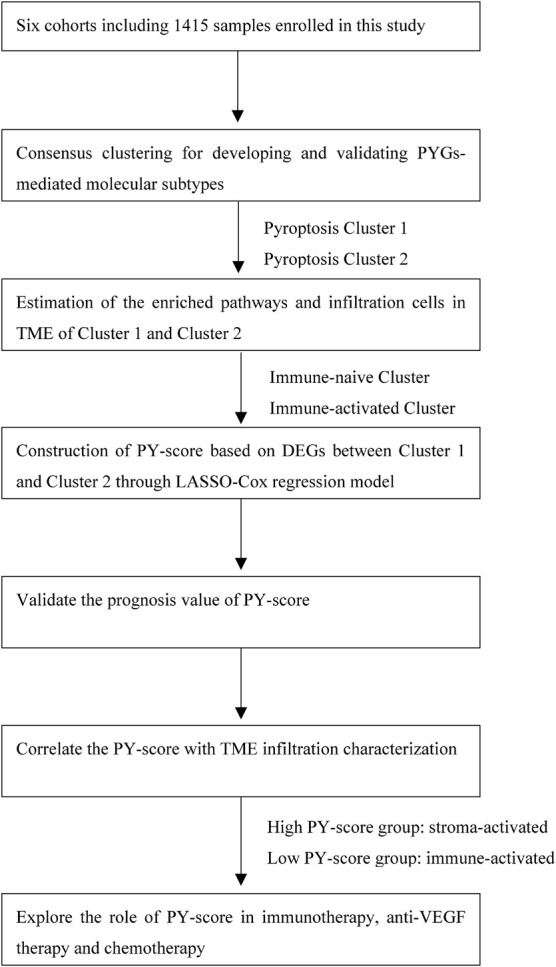
Overview of the study design and analysis of the pyroptosis-related genes in colon cancer patients.

The flowchart of our work is shown in Figure 1. RNA-sequencing data and clinical information were obtained from the GEO and TCGA databases. Overall, five eligible CC cohorts (GSE14333, GSE143985, GSE38832, GSE39582, and GSE140973) (Jorissen et al., 2009; Marisa et al., 2013; Tripathi et al., 2014; Shinto et al., 2020; Vangala et al., 2021) and The Cancer Genome Atlas—Colon Adenocarcinoma (TCGA-COAD) were included. The primary endpoint was recurrence of the tumor or death, and patients without survival information were removed from further analysis. The duration of follow-up in the meta-GEO cohorts (GSE143985, GSE38832, and GSE39582) was 10 years, while that in the GSE14333 cohort and TCGA-COAD cohort was 5 years due to the high proportions of censored patients. The GSE140973 cohort contained samples that received anti-EGFR therapy. PYGs were extracted from previous reviews and shown in Supplementary Table 1 (Man and Kanneganti, 2015; Wang and Yin, 2017; Karki and Kanneganti, 2019; Xia et al., 2019). Background adjustment and quantile normalization were performed for raw microarray gene expression data from Affymetrix through the “affy” and “simpleaffy” packages in R. As for the datasets from TCGA, the format of the RNA sequencing data was converted from fragments per kilobase per million reads (FPKM) into transcripts per million (TPM). Batch effects from the different GEO datasets were corrected through the “ComBat” algorithm in the “SVA” package. Data of somatic mutations in TCGA-COAD were also downloaded from TCGA dataset.

### Molecular Subtypes Based on Pyroptosis-Related Genes

Pyroptosis-related patterns were developed based on the aforementioned 10 PYGs through the “NMF” package. The non-negative expression matrix of the CC samples with the PYGs was factorized into a product of two non-negative matrices (*W* and *H*). The optimal clustering pattern was obtained based on proper cophenetic, dispersion, and silhouette coefficients from repeated performances.

### Identification of the Differentially Expressed Genes and Enriched Pathways

Before comparing the different groups (such as tumor vs. normal or cluster 1 vs. cluster 2) based on empirical Bayesian approach through the “limma” package, the messenger RNA (mRNA) expression data were normalized into *Z*-scores. Differentially expressed genes (DEGs) were then obtained by setting the corresponding adjusted *p*-values and the log2(fold change) values. Differentially enriched pathways could be identified based on the over-representation analysis method from the “clusterProfiler” package, which were divided into cellular component (CC), biological process (BP), and molecular function (MF) (Yu et al., 2012). Another way to detect subtle pathway activity changes over different groups is to use the “GSVA” package through a non-parametric and unsupervised method (Hanzelmann et al., 2013). The “c2.cp.kegg.v6.2” gene sets running for gene set variation analysis (GSVA) were downloaded from the MSigDB database.

### Estimation of Tumor Microenvironment Cell Infiltration and Immune Response Predictor

The TME in CC samples could be estimated through the Estimation of STromal and Immune cells in MAlignant Tumours using Expression data (ESTIMATE), which is a method that uses gene feature panels to infer the proportions of stromal and immune cells (Yoshihara et al., 2013). The immune score and the stroma score reflected the relative abundance of immune cells and stromal cells, respectively. The method used for evaluating more specific components of the TME is the single-sample gene set enrichment analysis (ssGSEA) algorithm. Based on special gene sets for marking some immune cells obtained from a recent study, we could quantify the relative abundance of 18 T-cell subsets in the TME of CC samples (Miao et al., 2020). Considering validation, we used another deconvolution approach, CIBERSORT,^1^ to calculate the relative abundance of 22 distinct immune cells through the gene expression profiles of the CC samples.

The immunophenoscore (IPS) is a newly identified biomarker for response to anti-CTLA4 and anti-PD1 therapy, which is a scoring scheme for quantifying the determinants of tumor immunogenicity through machine learning (Charoentong et al., 2017). We also downloaded the IPS of CC samples from TCGA cohort^2^ to estimate the response to immunotherapy.

### Generation of Pyroptosis-Related Gene Signature and Correlation Analysis

Differentially expressed genes between different pyroptosis-related patterns were screened using the “survfit” function of the “survival” package, and 98 prognosis-related DEGs with a significance level of *p* < 0.05 were identified for the subsequent construction of a gene signature. To avoid overfitting of the model, these genes were again screened based on least absolute shrinkage and selection operator (LASSO) estimation, and the optimal value for penalization coefficient lambda was chosen after running cross-validation likelihood (cvl) 1,000 times through the “glmnet” package (Tibshirani, 1997). Thus, the following equtation was established:


$$\begin{array}{rcl} \mathrm{Gene signature} & = & \;\sum \mathrm{Cox coefficient of gene Xi}\;\\ & & \;\times \;\mathrm{expression value of gene Xi}. \end{array}$$

Since those included in the gene signature were from the DEGs between two clusters with significantly different activities of pyroptosis, we speculated that these genes might be downstream or upstream of pyroptosis genes; thus, the final gene signature was termed the PY-score.

According to the method of maximally selected rank statistics, the optimal cutoff point was calculated to classify the CC patients into a low PY-score group and a high PY-score group using the “survminer” package. The survival difference between two groups can be visualized using the “ggsurvplot” function. The receiver operating characteristic (ROC) curve of the PY-scores was drawn using the “survivalROC” package. Quantification of the correlation used the “Spearman” method through the “cor.test” function in R.

### Statistical Analysis

In this study, we conducted statistical analyses using R-4.0.3. Student’s *t*-test was used to evaluate the statistical significance of normally distributed quantitative variables, while Wilcoxon’s rank-sum test was utilized to calculate the statistical significance of non-normally distributed quantitative variables. For comparisons of more than two groups, the Kruskal–Wallis test was used as the non-parametric method. All comparisons were two-sided, with an alpha level of 0.05.

## Results

### Genetic Variation of Pyroptosis-Related Genes in Colon Cancer

**FIGURE 2:**
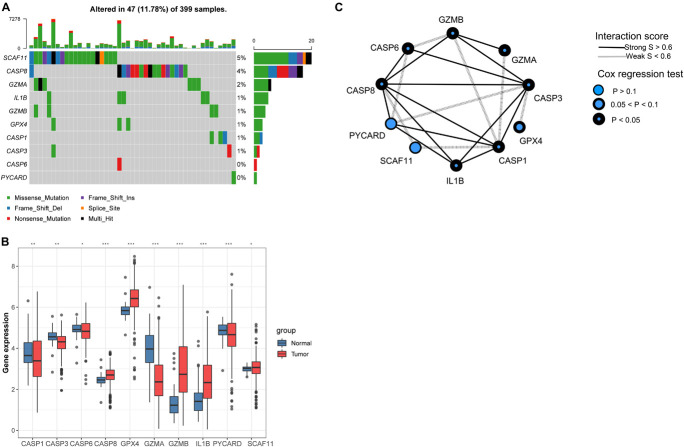
Expressions of 10 pyroptosis-related genes in colon cancer and normal tissues. **(A)** Mutation frequency of 10 pyroptosis-related genes in 399 colon cancer samples from The Cancer Genome Atlas—Colon Adenocarcinoma (TCGA-COAD) cohort. **(B)** Differences in the mRNA expression values of the 10 pyroptosis-related genes between the normal and colon cancer samples in TCGA-COAD cohort. *Asterisks* represent the statistical *p*-value (^*ns*^*p* > 0.05; *p < 0.05; **p < 0.01; ***p < 0.001). **(C)** Interaction of proteins in the 10 pyroptosis-related genes. *Lines connecting the pyroptosis-related genes* represent their interaction with each other. The *size of each circle* represents the prognostic effect of each regulator and scaled by the *p*-value.

Ten PYGs from the recent literature were identified in this study (Supplementary Table 1). Incidences of somatic mutations of the 10 PYGs in CC were calculated from TCGA-COAD cohort. Among 399 samples, 47 showed mutations of PYGs, in which *SCAF11* had the highest mutation frequency. The most common mutation type was missense mutation (Figure 2A). We also found prominent differences in the mRNA expressions of these genes between the normal and CC samples in TCGA-COAD cohort (Figure 2B), which indicated the vital role of PYGs in cancer occurrence and progression.

### Development of Pyroptosis-Related Genes-Mediated Molecular Patterns and Characteristics of Respective Tumor Microenvironment

Background adjustment and quantile normalization were performed for the mRNA expression matrices from the GSE1433, GSE143985, GSE38832, and GSE39582 cohorts to eliminate internal errors (Supplementary Figure 1). Through principal component analysis (PCA), the batch effect between the four cohorts was obvious (Supplementary Figure 2A), which was dealt with using the “ComBat” algorithm in the “SVA” package (Supplementary Figure 2B). Subsequently, we integrated three GEO datasets (GSE35982, GSE38832, and GSE143985) with available information on disease-free survival (DFS) into one meta-GEO cohort. The interaction between PYGs and their prognostic value is comprehensively presented in Figure 2C through a correlation analysis and a univariate Cox regression model in the meta-GEO cohort. The mRNA expression levels of most PYGs were significantly associated with the prognosis of patients, which indicated their potential in the genotyping of CC.

**FIGURE 3:**
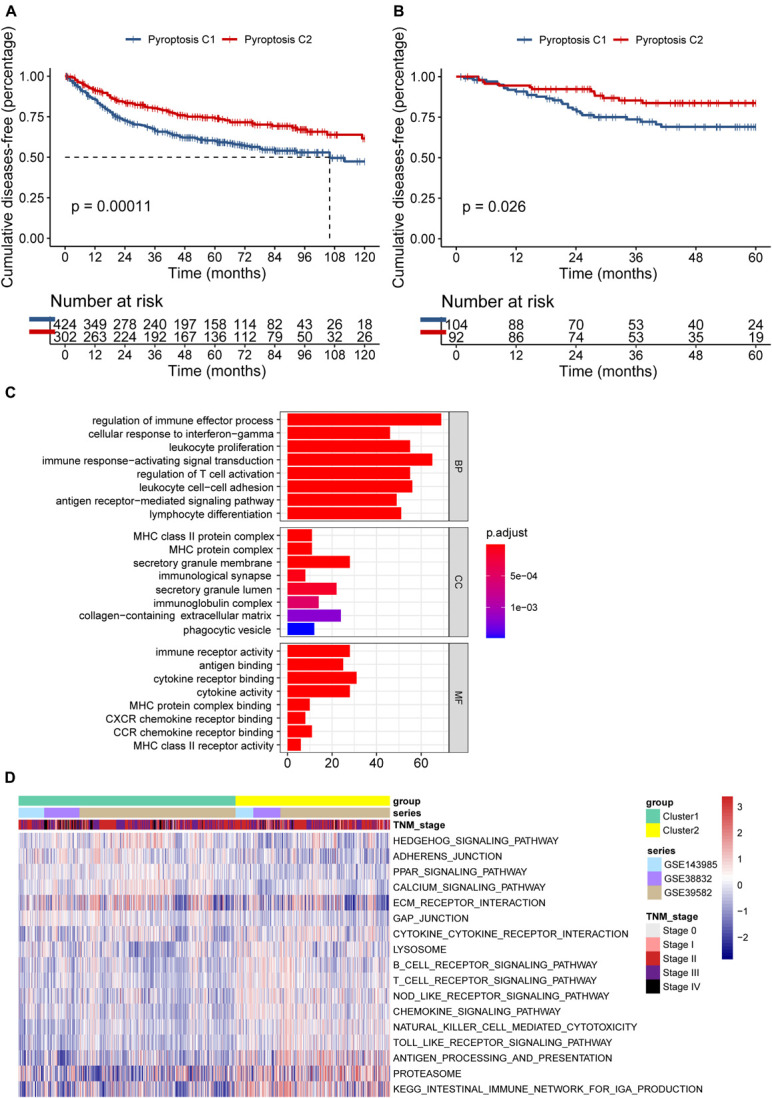
Differences in the prognosis and biological characteristics between two clusters. **(A)** Kaplan–Meier curves showed significant survival differences between pyroptosis C1 and pyroptosis C2 based on 726 colon cancer patients from the meta-GEO (Gene Expression Omnibus) cohort (*p* < 0.001). **(B)** Pyroptosis C2 also showed significantly better disease-free survival than did pyroptosis C1 in the GSE14333 cohort (*p* = 0.026). **(C)** Functional annotation of the differentially expressed pyroptosis-related genes between C1 and C2 using Gene Ontology (GO) enrichment analysis in the meta-GEO cohort. **(D)** Enrichment analysis using gene set variation analysis (GSVA) showing the activated biological pathways in distinct pyroptosis-related gene (PYG)-mediated molecular patterns in the meta-GEO cohort. The *red strip* represents the activated pathways and the *blue strip* refers to the inhibited pathways. C1, cluster 1; C2, cluster 2.

Seven hundred and 26 patients from the meta-GEO cohort served as the discovery group, which we divided into two distinct molecular clusters based on the unsupervised clustering method using the “NMF” package (Supplementary Figures 3A,B). Four hundred and 24 patients in cluster 1 (C1) had prominently worse prognosis than did the 302 patients from cluster 2 (C2) (*p* = 0.00011; Figure 3A). Besides, we validated the molecular patterns in CC patients from the GSE14333 cohort (Supplementary Figure 3C). Compared with patients in C1, those in C2 still showed a significant survival advantage (*p* = 0.026; Figure 3B), which confirmed the stability of the PYG-mediated molecular patterns.

To explore the potential biological characteristics of the PYG-mediated molecular patterns, we calculated 499 DEGs between two clusters using the “limma” package in the meta-GEO cohort (*p* < 0.0001 and | log-fold change| > 0.5). Gene Ontology (GO) enrichment analysis based on the “clusterProfiler” package is shown in Figure 3C. Enrichment of the different BPs, CC, and MF were all remarkably related to immunity (such as regulation of immune effector process), which indicated that PYG modification played an indubitable role in immune regulation in the TME. GSVA was also conducted for the meta-GEO cohort. From Figure 3D, it can be seen that the function of C2 was markedly enriched in immune-related pathways such as antigen processing and presentation and Toll-like receptor signaling pathway, while in C1, oncogenic pathways (such as Hedgehog signaling pathway) and stroma-related pathways [such as extracellular matrix (ECM) receptor interaction] were dramatically upregulated. These findings suggested the strong interactions between tumor tissues and the ECM in C1 and a large number of activated immune cells in the TME of C2.

**FIGURE 4:**
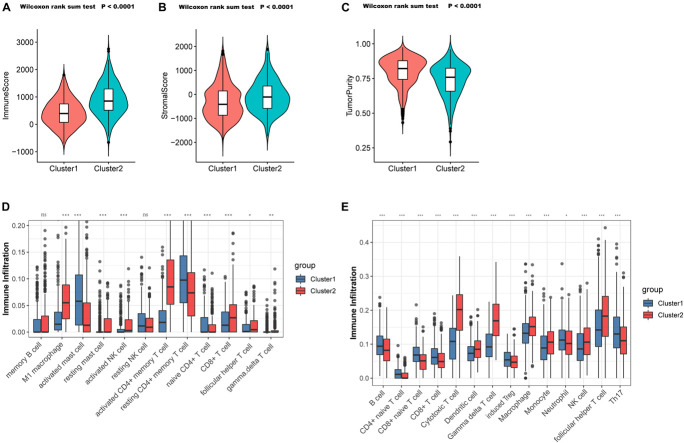
Tumor microenvironment (TME) cell infiltration characteristics in distinct PYG-mediated molecular patterns. **(A)** Samples in C1 showed significantly lower immune scores than did the samples in C2 in the meta-GEO (Gene Expression Omnibus) cohort (*p* < 0.0001). **(B)** Samples in C1 showed prominently higher stromal scores than did the samples in C2 in the meta-GEO cohort (*p* < 0.0001). **(C)** Samples in C1 showed markedly higher tumor purity than did the samples in C2 in the meta-GEO cohort (*p* < 0.0001). **(D,E)** Abundance of infiltrating immune cells in the two clusters based on the CIBERSORT platform **(D)** and the ImmuncellAI platform **(E)** in the meta-GEO cohort. *Asterisks* represent the statistical *p*-value (^*ns*^*p* > 0.05; *p < 0.05; **p < 0.01; ***p < 0.001). C1, cluster 1; C2, cluster 2; PYGs, pyroptosis-related genes.

To validate the aforementioned hypothesis, we subsequently tried to evaluate the TME of the two clusters more concretely. According to the “estimate” package, samples in C1 also exhibited markedly lower immune scores (*p* < 0.0001; Figure 4A) and stromal scores (*p* < 0.0001; Figure 4B), but higher tumor purity (*p* < 0.0001; Figure 4C) than did the samples in C2, which was consistent with previous findings. Through the CIBERSORT platform, we found that the microenvironment of the samples in C2 was filled with antitumor cells such as M1 macrophages, activated natural killer (NK) cells, gamma delta T cells, and follicular helper T cells, while resting or naive immune cells such as naive CD4^+^ T cells and resting CD4^+^ memory T cells dominated in the samples from C1 (Figure 4D). In the ImmuncellAI platform, we also observed the abundance of resting or naive immune cells in C1 (such as naive CD4^+^ and CD8^+^ T cells) and extensive activated antitumor cells in C2 (cytotoxic T cells and gamma delta T cells) (Figure 4E). Thus, it was confirmed that PYG-mediated molecular patterns indicate not only different survival outcomes but also different TMEs.

### Generation of the Pyroptosis-Score and Functional Annotation

Although the PYG-mediated molecular subtypes showed a significantly different survival advantage, there was no quantitative method to determine which cluster an individual patient belonged to. Thus, a scoring system to accurately predict the molecular subtype in an individual patient is urgently needed. Firstly, we screened 98 DEGs that were significantly associated with prognosis from the 499 DEGs between the two clusters (*p* < 0.05). Subsequently, six genes were selected for the final scoring system through the application of the LASSO–Cox regression model in the meta-GEO cohort (Supplementary Figure 4).


$$\begin{array}{rcl} \mathrm{Gene signature} & = & \mathrm{APOL}6\;\times \;\left(-0.20430753\right)+C5\mathrm{AR}1\;\\ & & \;\times \;0.05298134+\mathrm{CXCL}1\;\times \;\left(-0.01986885\right)\\ & & \;+\mathrm{CXCL}13\;\times \;\left(-0.04158380\right)+\mathrm{ENPP}2\;\\ & & \;\times \;0.04349674+\mathrm{NLRC}5\;\times \;\left(-0.03656359\right). \end{array}$$

Since the six genes were obtained from the differential expression analysis between clusters 1 and 2, which had prominently different pyroptosis activities, we inferred that the six genes might be downstream or upstream of the pyroptosis genes; thus, we termed the gene signature as the PY-score.

**FIGURE 5:**
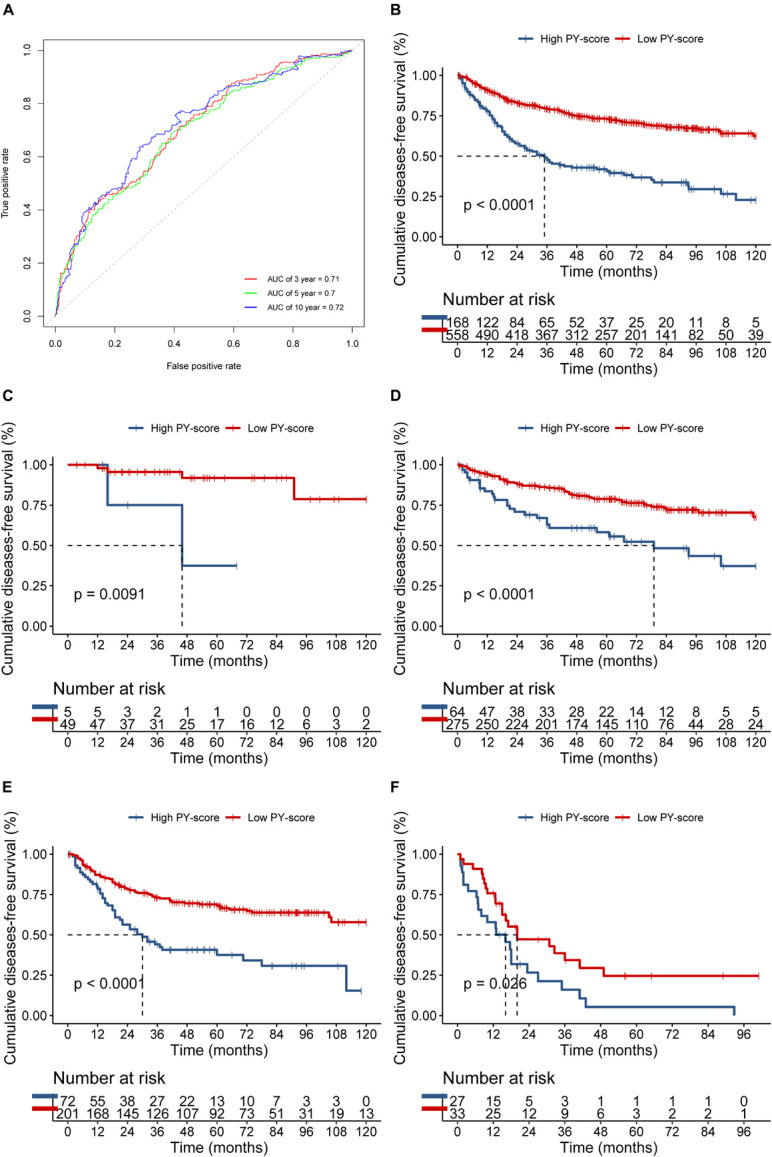
Role of the pyroptosis score (PY-score) in predicting the survival of colon cancer patients in the meta-GEO (Gene Expression Omnibus) cohort. **(A)** Predictive value of the PY-score in colon cancer patients from the meta-GEO cohort. **(B)** Survival analyses for patients in the low (558 cases) and high (168 cases) PY-score groups in the meta-GEO cohort using Kaplan–Meier curves (*p* < 0.001). **(C–E)** Survival analyses for tumor node metastasis (TNM) stage 0 and I patients in the low (49 cases) and high (5 cases) PY-score groups (*p* = 0.0091) **(C)**, TNM stage II patients in the low (275 cases) and high (64 cases) PY-score groups (*p* < 0.001) **(D)**, TNM stage III patients from the low (201 cases) and high (72 cases) PY-score groups (*p* < 0.001) **(E)**, and TNM stage IV patients from the low (33 cases) and high (27 cases) PY-score groups (*p* = 0.026) **(F)** from the meta-GEO cohort using Kaplan–Meier curves.

The PY-score showed good performance in the survival prediction of CC patients, with a 5-year area under the ROC curve (AUC) of 0.70 and a 10-year AUC of 0.72 (Figure 5A). Based on the PY-score, we divided the CC patients into two groups and found prominent survival differences between them (*p* < 0.0001; Figure 5B). Patients with a low PY-score also exhibited significant survival advantage than did patients with a high PY-score in different TNM stages (Figures 5C–F), which indicated that the PY-score could further stratify patients based on traditional clinical stages.

**FIGURE 6:**
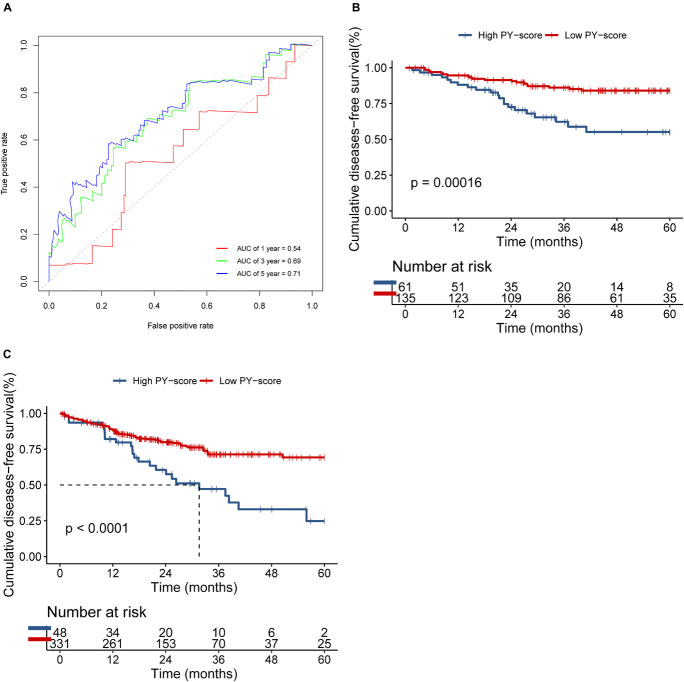
Validation of the role of the pyroptosis score (PY-score) in predicting the survival of colon cancer patients through the GSE14333 and TCGA-COAD cohorts. **(A)** Predictive value of the PY-score in colon cancer patients from the GSE14333 cohort. **(B,C)** Survival analyses for patients from the low (135 cases) and high (61 cases) PY-score groups in the GSE14333 cohort (*p* < 0.001) and for patients from the low (331 cases) and high (48 cases) PY-score groups in TCGA-COAD cohort (*p* < 0.001) **(C)** using Kaplan–Meier curves.

We next verified the constructed PY-score in the GSE14333 cohort. The PY-score also exhibited potential prognostic predictive value (5-year AUC = 0.71; Figure 6A), and a low PY-score predicted a prominent survival benefit (*p* < 0.001; Figure 6B). To further validate the stability of the PY-score, we evaluated the correlation between the PY-score and the prognosis of patients in the cohort from TCGA. Although a significant survival difference between the low PY-score group and the high PY-score group was observed (*p* < 0.0001; Figure 6C), the performance of the PY-score in precisely predicting prognosis was unsatisfactory (5-year AUC = 0.601), which suggested potential differences between the RNA sequencing platforms. Thus, the above results indicated that the PY-score could represent the PYG-mediated molecular patterns to predict the prognosis of CC patients though microarray expression data.

**FIGURE 7:**
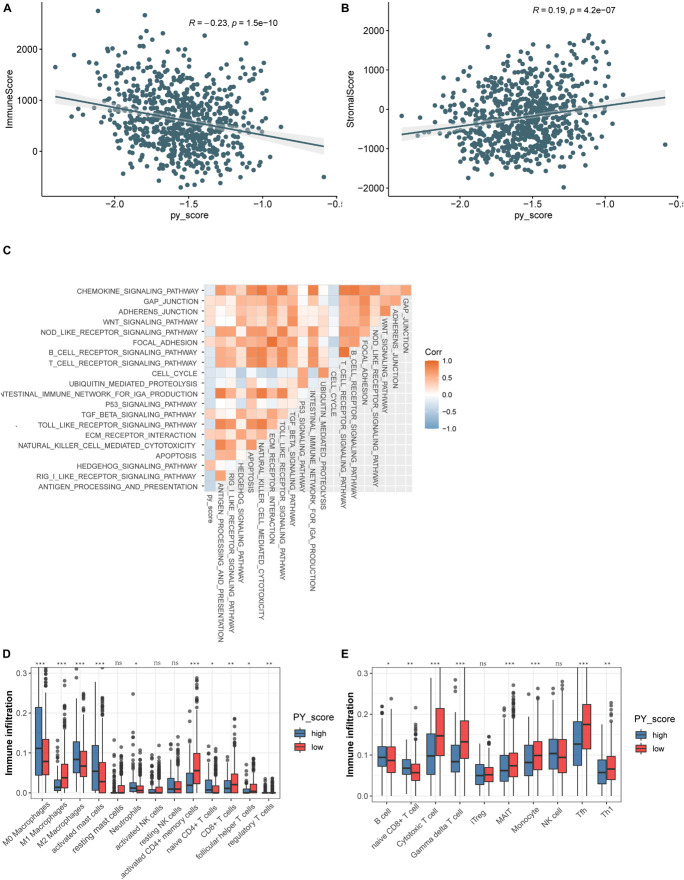
Function annotation of the pyroptosis score (PY-score). **(A,B)** Relationships between the immune score and PY-score (Spearman’s test: *p* < 0.0001) **(A)** and the between the stromal score and PY-score (Spearman’s test: *p* < 0.0001) **(B)** in the meta-GEO cohort. **(C)** Correlations between the PY-score and the known gene signatures in the meta-GEO cohort using Spearman’s analysis. A negative correlation is marked with *blue* and a positive correlation with *orange*. **(D)** Abundance of infiltrating immune cells in the high and low PY-score groups based on the CIBERSORT platform in the meta-GEO cohort. *Asterisks* represent the statistical *p*-value (^*ns*^*p* > 0.05; *p < 0.05; **p < 0.01; ***p < 0.001). **(E)** Abundance of infiltrating immune cells in the high and low PY-score groups based on the ImmuncellAI platform in the meta-GEO cohort. *Asterisks* represent the statistical *p*-value (^*ns*^*p* > 0.05; *p < 0.05; **p < 0.01; ***p < 0.001).

To investigate the biological function of the different PY-scores, we calculated the immune score and the stromal score of individual patients. A significant negative correlation was found between the PY-score and the immune score (*p* < 0.0001; Figure 7A), while the PY-score was positively associated with the stromal score (*p* < 0.0001; Figure 7B). To better illustrate the characteristics of the PY-score, we tested its correlation with the known signatures based on Spearman’s analysis (Figure 7C). A positive correlation was also found between the PY-score and the stroma-related pathways such as ECM receptor interaction and adherens junction, while a negative correlation was found between the PY-score and the immune-related pathways such as antigen processing and presentation and NK cell-mediated cytotoxicity. Through the CIBERSORT platform, we observed more abundant M1 macrophages in the low PY-score group, while more M0 and M2 macrophages were found in the high PY-score group (Figure 7D). Based on the ImmuncellAI platform, it was also found that patients with a low PY-score had more activated immune cells, such as cytotoxic T cells and gamma delta T cells, while patients with a high-PY score had naive CD8^+^ T cells (Figure 7E). Thus, the above results strongly suggested that a low PY-score was significantly associated with immune activation and that a high PY-score was correlated with stromal activation, which indicated that the PY-score could not only predict the prognosis of an individual patient but also evaluate the characterization of the immune infiltration.

### Pyroptosis-Score in the Role of Immunotherapy, Anti-epidermal Growth Factor Receptor Therapy, and Chemotherapy

**FIGURE 8:**
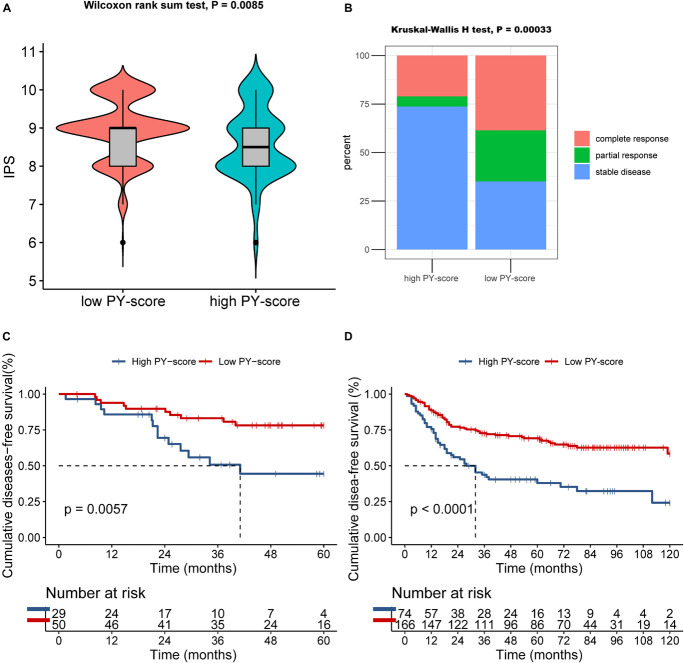
Role of the pyroptosis score (PY-score) in immune checkpoint inhibitor (ICI) treatment, anti-epidermal growth factor receptor (EGFR) therapy, and chemotherapy. **(A)** Samples in the high PY-score group showed a significantly lower immunophenoscore (IPS) than did the samples in the low PY-score group in the meta-GEO cohort (*p* = 0.0085). **(B)** Fraction of samples with clinical response to anti-EGFR therapy in the low and high PY-score groups of the GSE140973 cohort (*p* < 0.001). **(C,D)** Survival analyses for patients who received chemotherapy from the low (558 cases) and high (168 cases) PY-score groups in the GSE14333 (*p* < 0.001) **(C)** and from the low (558 cases) and high (168 cases) PY-score groups in the meta-GEO cohort (*p* = 0.0057) **(D)** using Kaplan–Meier curves.

The presentation of ICI treatment undoubtedly marked a great breakthrough toward antitumor therapy. In addition to well-known biomarkers such as programmed death-ligand 1 (PD-L1), the newly identified IPS has also been widely used to supervise the immune response (Charoentong et al., 2017). In TCGA cohort, we also observed that the IPS was prominently decreased in patients with a high PY-score (*p* = 0.0085; Figure 8A), which indirectly reflected the strong connection between the PY-score and response to immunotherapy.

Besides ICI treatment, the role of anti-EGFR therapy could also not be ignored in CC patients. Thus, we subsequently investigated whether the PY-score could predict responses to anti-EGFR therapy. In the GSE140973 cohort, significant therapeutic benefits were found in CC samples with a low PY-score (*p* < 0.001; Figure 8B).

Although diverse treatment options have emerged in the past years, chemotherapy still is the first choice for CC patients in the advanced stages. Therefore, exploration was also needed of the impact of the PY-score in the chemotherapy response of CC patients. Firstly, in the meta-GEO cohort, patients with a low PY-score presented significantly prolonged survival than did patients with a high PY-score, all of whom received chemotherapy (*p* < 0.0001; Figure 8D). Next, the marked survival benefits were confirmed in the low PY-score group when compared with the high PY-score group among patients who received chemotherapy from the GSE14333 cohort (*p* = 0.0057; Figure 8C).

Taken together, our findings indicated that the PY-score correlated with the responses to immunotherapy, anti-EGFR therapy, and chemotherapy. In addition, the PY-score can further predict survival among CC patients.

## Discussion

Emerging evidence has supported the vital role of pyroptosis in the occurrence, progression, chemosensitivity, and inflammation of CC. However, the overall impact of PYGs on the prognosis and the TME characteristics of CC has not been comprehensively clarified. Therefore, construction of distinct molecular subtypes based on PYGs might not only predict patient survival but also provide insights into PYG-mediated immune response and, thus, guide more appropriate therapeutic strategies.

In this research, we developed and validated two distinct PYG-mediated molecular subtypes with significantly different prognosis and TME characteristics in the meta-GEO and GSE14333 cohorts. Samples in C1 showed prominent survival advantage with the enrichment of immune-related pathways and the infiltration of activated antitumor immune cells. On the other hand, samples in C2 showed relatively poor prognosis with the enrichment of oncogenic- or stromal-related pathways and the infiltration of naive immune cells. It was reported that NK cells and cytotoxic T lymphocytes could kill gasdermin B (GSDMB)-positive cells through lymphocyte-derived granzyme A (GZMA). Thus, different immune infiltrations were found between the two PYG-mediated molecular subtypes. Increasing evidence also demonstrated that cancer progression strongly depended on the components of the TME: the ratio of CD206^+^ tumor-associated macrophages/CD68^+^ tumor-associated macrophages, NK cells, cytotoxic T cells, and gamma delta T cells served as independent risk factors for patients with CC (Janakiram et al., 2016; Feng et al., 2019; Mlecnik et al., 2020; Lu et al., 2021). A previous research showed significantly poor prognosis in CC patients with high stromal components (Eriksen et al., 2018). Stromal cells such as cancer-associated fibroblasts and differentiated perivascular-like cells also exhibited obvious correlations with cytotoxic T-cell dysfunction and exclusion, respectively (Wu et al., 2020), which was consistent with the immune-excluded and stromal-activated status in C2.

To precisely predict the prognosis of individual CC patients, we established the pyroptosis scoring system based on the LASSO–Cox regression model. Unlike traditional ways of model construction, the LASSO method could retain as many variables as possible, but avoids overfitting of the models through L1 regularization. Some studies applied PCA to construct a gene signature (Sotiriou et al., 2006; Zeng et al., 2019). Although this method showed superiority in focusing the score with the largest block of well-correlated genes in the gene set, the excessively specific scoring method might limit the robustness and its application. In this study, the PY-score showed good performance in predicting the survival of patients both in the meta-GEO cohort and the GSE14333 cohort, while the performance was not satisfactory in TCGA-COAD cohort. This result might be associated with the obvious batch effect between the different platforms of microarray data and RNA sequencing data, which is a common problem in the construction of a prognostic model through mRNA expression data. Thus, we recommend that it might be more proper to apply the PY-score using microarray data.

Patients were divided into a high PY-score group and a low PY-score group. Besides the prominently different prognosis, the distinct TME of the two groups was also observed based on reverse correlations with the immune scores and estimation of different immune cells. Similar to the PYG-mediated molecular subtypes, samples in the high PY-score group exhibited an immune-excluded condition with the infiltration of M2 macrophages and naive CD8^+^ T cells, while samples in the low PY-score group exhibited an immune-activated condition with the infiltration of M1 macrophages and cytotoxic T cells. Increasing evidence confirmed that the regulation of the TME plays a crucial role in response to antitumor therapy. A recent research has found that transforming growth factor beta (TGF-β) signaling in the TME suppressed the antitumor effects of the diverse immune cell subtypes (such as T cells and NK cells) and then limited the response to immunotherapy (Derynck et al., 2021). We also observed a significantly positive correlation between the PY-score and enrichment of the TGF-β signaling pathway in this study. It was revealed that hot tumors, which display a high degree of T-cell infiltration, tended to show good response to ICI therapy or combination therapy, while the response of cold tumors, which lack a preexisting immune response, to immunotherapy tended to be unsatisfactory (Galon and Bruni, 2019). M2 macrophages were reported to antagonize the antitumor activity of chemotherapy by orchestrating a tumor-promoting or tissue repair response (Mantovani et al., 2017). The re-plasticity of the TME, which is characterized by the infiltration of tumor-associated immune cells and the upregulation of immune checkpoint-related proteins, was also responsible for the resistance to anti-EGFR drugs and treatment failure (Misale et al., 2014; Woolston et al., 2019). Thus, the reason for the PY-score being able to predict responses to immunotherapy, anti-EGFR therapy, and chemotherapy might be attributed to its ability to differentiate the two distinct TMEs.

Some limitations must be recognized. Firstly, due to the absence of ICI-based CC datasets, we could only calculate the correlation between the PY-score and IPS and then estimate the response to ICI. Associated research studies are needed to confirm the effect of the PY-score. Besides, all the datasets for the development and validation of the PY-score were retrospective; thus, a prospective cohort of CC patients is also needed.

In this research, we comprehensively constructed the PY-score among 1,415 CC samples that could predict patient survival and responses to diverse therapeutic strategies based on distinct TME cell-infiltrating characteristics. This integrated analysis might provide insights for understanding tumor immunity and individualized precise therapy.

## Data Availability Statement

The datasets presented in this study can be found in online repositories. The names of the repository/repositories and accession number(s) can be found in the article/Supplementary Material.

## Author Contributions

CC designed the study, obtained the funding, and supervised the study. JR collected the data, performed the analysis, and prepared the figures. WL wrote the manuscript. All authors contributed to the article and approved the submitted version.

## Conflict of Interest

The authors declare that the research was conducted in the absence of any commercial or financial relationships that could be construed as a potential conflict of interest.

## Publisher’s Note

All claims expressed in this article are solely those of the authors and do not necessarily represent those of their affiliated organizations, or those of the publisher, the editors and the reviewers. Any product that may be evaluated in this article, or claim that may be made by its manufacturer, is not guaranteed or endorsed by the publisher.

## Footnotes

1
http://cibersort.stanford.edu/

2
https://tcia.at/

## Abbreviations

PYGs: pyroptosis-related genes
C1: cluster 1
C2: cluster 2
TME: tumor microenvironment
CC: colon cancer
TNM: tumor node metastasis
GEO: Gene Expression Omnibus
TCGA: The Cancer Genome Atlas
TCGA-COAD: The Cancer Genome Atlas—Colon Adenocarcinoma
DEGs: differentially expressed genes.
