# Supplementary material for: Pyroptosis-Mediated Molecular Subtypes and Tumor Microenvironment Infiltration Characterization in Colon Cancer
Source: Front Cell Dev Biol. 2021 Nov 10;9:766503. doi: 10.3389/fcell.2021.766503 (PMC8631352; doi:10.3389/fcell.2021.766503)
Supplement: Supplementary file 1 [file Data_Sheet_1.docx]

Supplementary Material

# Supplementary Data

None

# Supplementary Figures and Tables

## Supplementary Figures


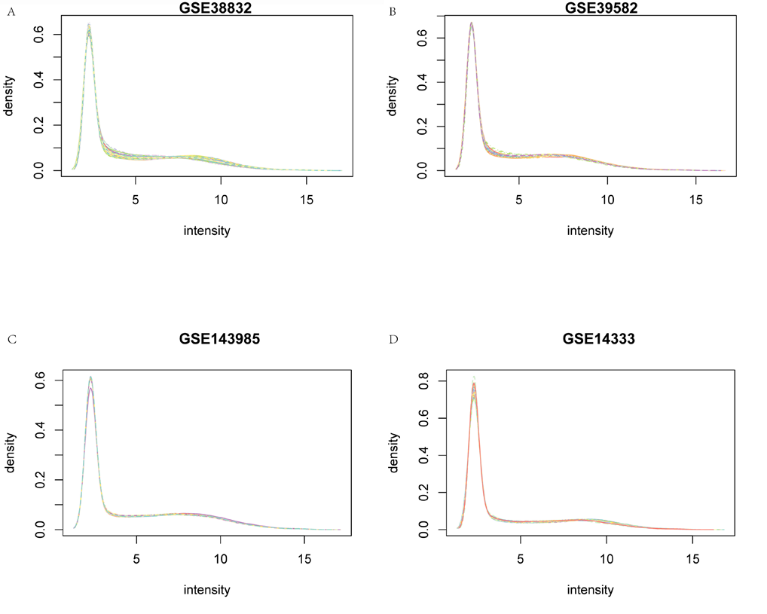


**Supplementary Figure 1. The preprocessing of mRNA expression data from GEO datasets.**

(A) The curves of expression data of individual sample in GSE38832 cohort almost coincided, which indicated the elimination of internal errors.

(B) The curves of expression data of individual sample in GSE39582 cohort almost coincided, which indicated the elimination of internal errors.

(C) The curves of expression data of individual sample in GSE143985 cohort almost coincided, which indicated the elimination of internal errors.

(D) The curves of expression data of individual sample in GSE14333 cohort almost coincided, which indicated the elimination of internal errors.


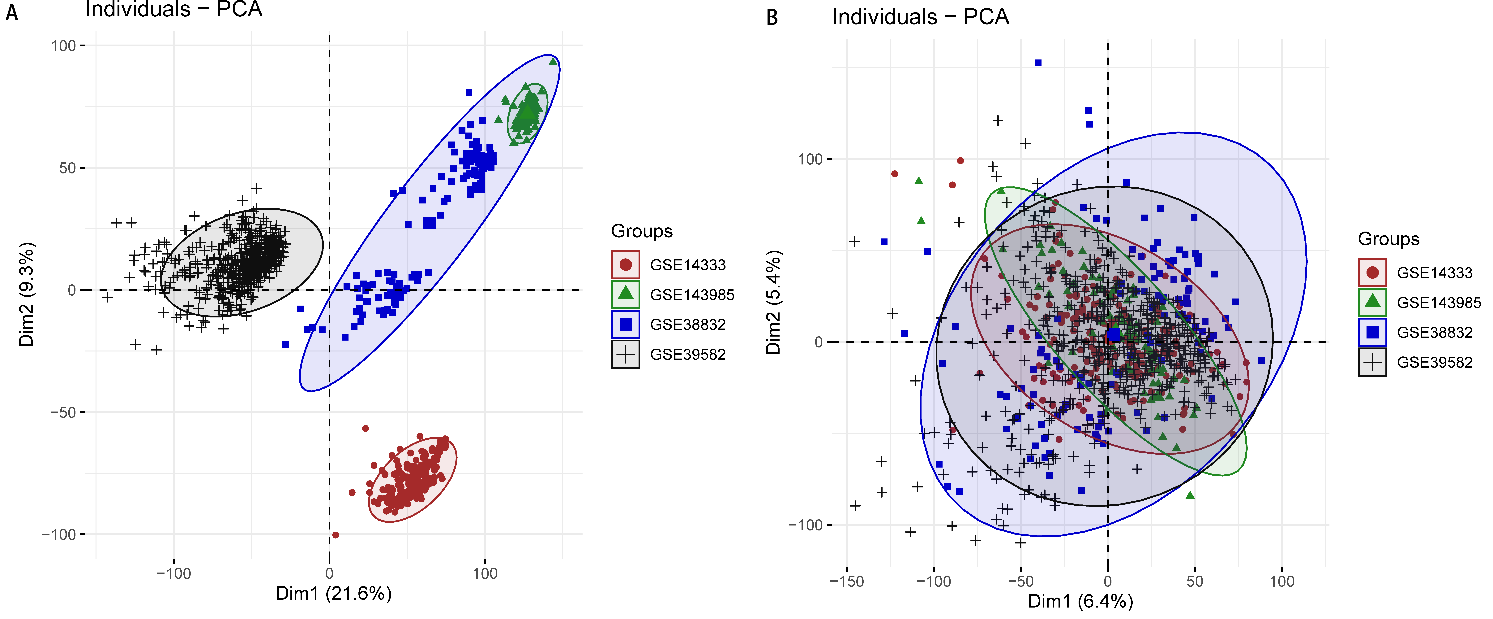


**Supplementary Figure 2. PCA analysis showed the batch effect between cohorts.**

(A) The PCA analysis showed obvious batch efforts before the application of “Combat” function;

(B) The PCA analysis showed less batch efforts after the application of “Combat” function;


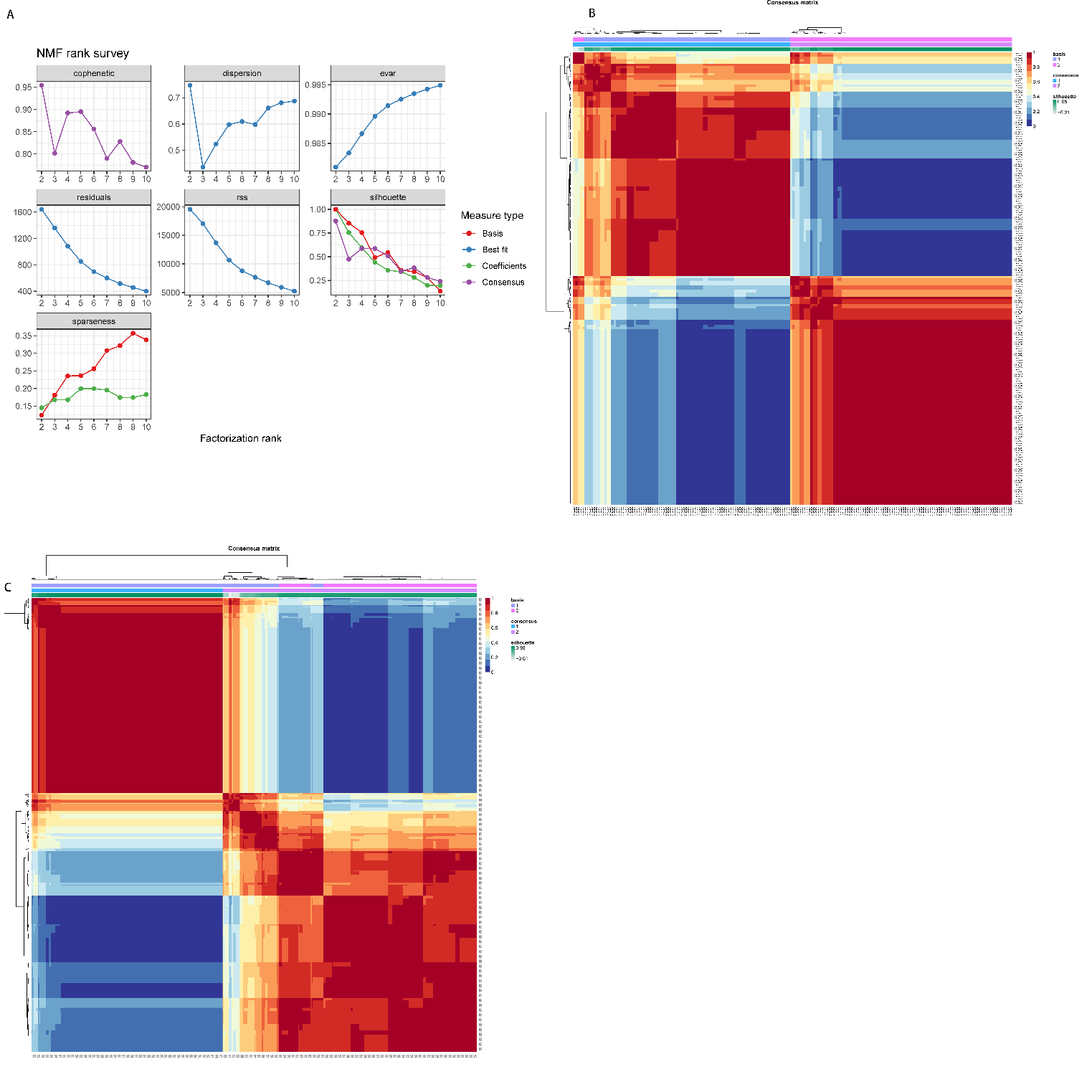


**Supplementary Figure 3. Molecular subtypes of colon cancer patients based on “NMF” package.**

(A) Based on cophenetic, dispersion and silhouette coefficients, it was proper to divide samples from meta-GEO cohort into 2 clusters.

(B) The consensus score matrix of all samples when k = 2 in meta-GEO cohort.

(C) The consensus score matrix of all samples when k = 2 in GSE14333 cohort.

**
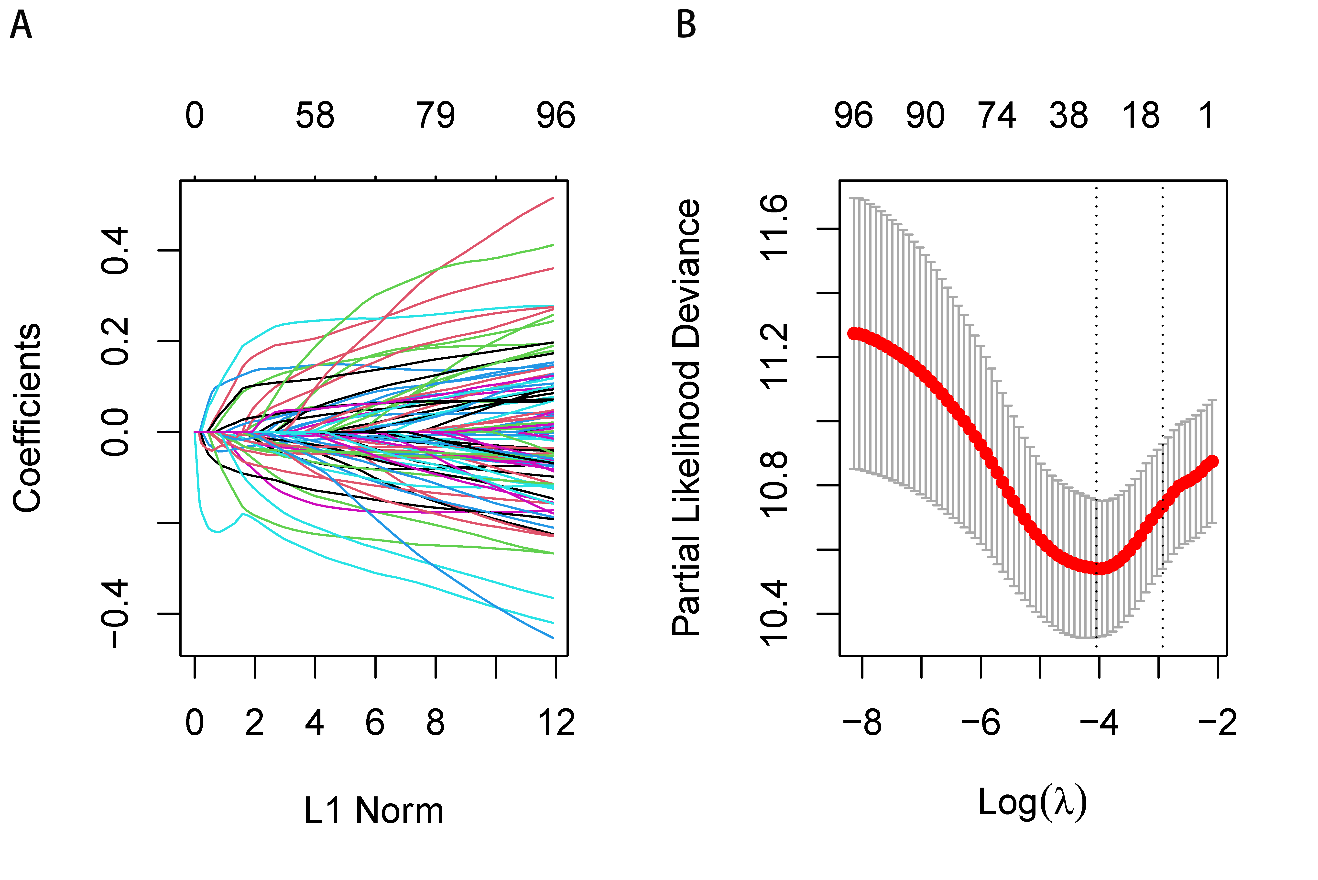
**

**Supplementary Figure 4. LASSO-Cox model based on pyroptosis-related genes.**

(A) LASSO coefficient profiles of the 98 genes of high prognostic value.

(B) The optimal values of the penalty parameter were determined by 10-fold cross-validation.

## Supplementary tables

**Supplementary table 1: 10 pyroptosis-related genes which were used to subtype the colon cancer patients.**

|  | gene_id |
| --- | --- |
| 1. | GPX4 |
| 2. | SCAF11 |
| 3. | CASP6 |
| 4. | CASP8 |
| 5. | CASP1 |
| 6. | PYCARD |
| 7. | IL1B |
| 8. | GZMA |
| 9. | GZMB |
| 10. | CASP3 |
